# Social cognition in cervical dystonia

**DOI:** 10.1016/j.prdoa.2023.100217

**Published:** 2023-09-09

**Authors:** Laura Mahady, Jessica White, Shameer Rafee, Siew-Mei Yap, Sean O'Riordan, Michael Hutchinson, Patricia Gough, Fiadhnait O'Keeffe

**Affiliations:** a School of Psychology, University College Dublin, Belfield, Dublin 4, Ireland; b Department of Neurology, St Vincent's University Hospital, Elm Park, Dublin 4, Ireland; c School of Medicine, University College Dublin, Belfield, Dublin 4, Ireland

**Keywords:** Social cognition, Cervical dystonia, Cognition, Non-motor symptoms, Movement disorders

## Abstract

**Background:**

Whilst traditionally considered a movement disorder, it is now generally accepted that cervical dystonia (CD) presents with additional non-motor symptoms which significantly impact quality of life. Our study primarily aimed to explore social cognition and levels of psychological distress in individuals with CD, in comparison to age- and sex-matched healthy controls.

**Methods:**

20 participants with CD attending a specialist movement disorders clinic were recruited. 20 age and sex matched neurologically healthy controls were recruited in parallel. Participants completed the Hospital Anxiety and Depression Scale, and two novel social cognition tasks: The Cambridge Mindreading Face-Voice Battery (CAFMB) and the Edinburgh Social Cognition Test (ESCoT).

**Results:**

Participants with CD exhibited poorer complex emotion recognition abilities for visual and auditory stimuli, compared to controls on the CAFMB task. Participants with CD did not differ significantly from controls on performance on cognitive or affective Theory of Mind tasks, or interpersonal or intrapersonal understanding of social norms, as measured by the ESCoT. The proportion of depressive symptoms was significantly higher for participants with CD than controls. 40% of participants with CD reported clinically elevated depressive symptoms, and 60% reported clinically elevated anxiety. Poorer understanding of emotional facial expressions was associated with higher levels of depression in the CD group.

**Conclusions:**

Significant between-group differences between participants with CD and controls suggests socio-cognitive dysfunction is an important aspect of the non-motor syndrome of CD. Findings highlight the need for assessment of and intervention for both social cognitive difficulties and psychological distress in individuals with CD.

## Highlights

• Socio-cognitive deficits are a key aspect of the non-motor syndrome of cervical dystonia.
• Individuals with CD demonstrated poorer recognition of complex emotions compared to controls.
• Our study used sensitive measures of social cognition, novel to the CD literature.
• 40% of participants with cervical dystonia report clinically elevated depressive symptoms.
• Higher levels of depression were associated with poorer performance on understanding of emotional facial expressions in CD.

## 1 Introduction

Traditionally considered a movement disorder, it is now generally accepted that cervical dystonia (CD) presents with additional non-motor symptoms [1]. Non-motor symptoms may negatively influence the quality of life of individuals with CD more than motor symptomology [1], [2], [3]. Such symptoms include psychological distress [4], and difficulties in various cognitive domains [2], including social cognition (SC) [6], [7].

Socio-cognitive domains reported to be affected in individuals with CD include emotion recognition and theory of mind (ToM) abilities [6], [7]. Isolated deficits have been reported in identifying facial expressions of disgust [8] and fear [9] and auditory expressions of anger [10] in individuals with CD. In a case-control cohort study, participants with CD performed significantly worse in labelling emotions for both visual and auditory stimuli, compared to controls [6] and normative data [7].

ToM, the capacity to understand the thoughts, emotions, and beliefs of others [11], is considered to involve cognitive and affective elements. Affective ToM is commonly measured using static media depicting emotional states; such as the Reading The Mind in the Eyes Task (RMET) [12] which requires participants to infer emotions based upon photographs of the eye region. Cognitive ToM is generally measured using false-belief tasks, where the participant must separate a character’s and their own perspective of an event. Poorer performance by participants with CD than controls on a measure of cognitive ToM was reported, primarily for those presenting with tremor. Comparable, less significant difficulties in affective ToM were reported for participants with and without tremor [13]. Individuals with CD performed significantly worse than controls on the Faux Pas (FP) test [14], with difficulties primarily in the cognitive domain [5]. A further study indicated that individuals with CD were unimpaired in affective ToM using the RMET [6], [7]. Individuals with CD performed in the below average range in identifying “Faux Pas” social interactions; which was associated with poorer quality of life [15]. One study [5] reported that over 95% of participants with CD were unimpaired on their understanding of implicit social rules. Recognition of complex emotional and mental states [11] beyond basic emotions (e.g. happy) [16] has not been directly studied in CD.

Conflicting results to date could be attributed to heterogeneity in CD populations (comorbidities [5], age [17], presence of tremor [13]), variability in study design and the myriad of SC measures used which vary in terms of stimuli, difficulty, and reliability**.** There are few validated measures of SC for neurological conditions [18]. Further, many SC tasks are affected by broader cognitive abilities and demographic characteristics. Many require comprehension of complex verbal narratives, the FP test is affected by age [19], and RMET performance is predicted by verbal comprehension ability [20]. Finally, many common SC tasks have been criticized as lacking ecological validity and may not reflect the functional application of SC abilities [21]. Most measures utilize photographs to measure emotion recognition; tasks which utilise moving stimuli and incorporate audio, such as the Cambridge Mindreading Face and Voice Battery (CAMFB) [22], may provide a more valid depiction of emotion.

Regarding ToM, many common measures present static, verbal narratives; measures which utilize dynamic visual information portraying a social interaction are more ecologically valid [21]. The Edinburgh Social Cognition Test (ESCoT) [15], utilises short animations to measure affective and cognitive ToM, and social norm understanding. Whilst other ecologically valid measures have also been developed, the ESCoT gathers qualitative information which may more finely capture individual differences in mental state understanding. Performance is not affected by verbal comprehension, perceptual reasoning [20] or executive abilities in healthy adults [23] and it has been validated in another neurological population (brain injury) [18]. Thus, the ESCoT may provide a preferable measure of higher order SC in CD.

Our study primarily aimed to explore SC in individuals with CD, in comparison to age- and sex-matched healthy controls (HCs), using SC measures novel to the CD literature; the CAMFB [22] and the ESCoT [20]. A secondary aim was to examine levels of psychological distress in this cohort of individuals with CD, compared to controls. It was hypothesized that participants with CD would exhibit poorer performance on SC measures and report higher levels of clinically significant psychological distress symptoms than controls.

## 2 Method

### 2.1 Study population

Participants with CD, satisfying standard diagnostic criteria, attending a movement disorders clinic at the host institution, a tertiary level hospital, were invited to participate. Participants were recruited and assessed from January to December 2022. Exclusion criteria included significant psychiatric illness and learning disability.

### 2.2 Healthy controls

Age- and sex-matched HCs were recruited in parallel using via convenience sampling. HCs were excluded in line with participant exclusion criteria, with the additional exclusion criterion of having a neurological condition.

The study was reviewed and approved by the local Research Ethics Committee. All participants gave written informed consent prior to their participation.

## 3 Materials

### 3.1 Demographic data was collected for all participants

#### 3.1.1 Hospital anxiety and depression Scale

Mood was assessed using the Hospital Anxiety and Depression Scale (HADS) [24], a self-report measure of depression and anxiety symptoms validated for hospital outpatient settings [24]. The measure provides an anxiety (HADS-A) and depression (HADS-D) score, with higher scores indicating higher levels of psychological distress. Subscale scores of 8 or greater indicate a clinically significant level of symptoms [25].

#### 3.1.2 Social Cognition measures

Social Cognition was examined using two separate measures: The Cambridge Mindreading Face-Voice Battery (CAMFB) [22] and the Edinburgh Social Cognition Test (ESCoT) [20].

The CAMFB assesses recognition of 20 complex emotions and mental states using visual and audio stimuli [22]. Participants are presented with 50 silent 3–5 s video clips of facial expressions, 50 audio clips of expressioned voices and asked to identify which of four adjectives best describe the person in the presented stimulus’ emotions. Participants may score between 0 and 50 on each subscale (CAM-Faces and CAM-Voices) with higher scores indicating better recognition of emotion and mental states (minimum possible score: 0, maximum possible score: 100).

The ESCoT [20] is an animated test that assesses four domains of higher order SC; cognitive ToM, affective ToM, and interpersonal and intrapersonal understanding of social norms. Participants are shown 11 animations: 1 practice animation, 5 portraying a social norm violation and 5 appropriate interactions. Participants are then asked questions regarding the interaction reflecting each of the four socio-cognitive domains and are prompted once if their answer lacks information. Higher scores indicate greater socio-cognitive performance (maximum score of 120) [20].

### 3.2 Statistical analysis

Descriptive statistics and between-groups comparisons were computed using JASP (2023) open-source statistical software. Participants’ scores for symptoms of anxiety (HADS-A) and depression (HADS-D) were grouped into clinically significant (scores > 8) or sub-clinical (scores 8 or greater) cases [25]. Two chi-square tests of independence were computed to compare the proportion of clinically significant cases across both groups.

T-tests were conducted to examine between-group differences for subscales (CAM:Voices, Cam:Faces) and total CAMFB scores. Non-parametric Mann-Whitney U tests were conducted to examine between-group differences for ESCoT: Cognitive ToM scores. A MANOVA was conducted to compare groups on the remaining ESCoT subscales.

## 4 Results

### 4.1 Demographics

20 participants with CD and 20 HCs participated in the study. Participants in each group were matched for age (CD: 51.00 ± 10.77 years, range: 24–66; HC: 49.15 ± 11.99 years, range: 23–64, p = 0.611), years of education (CD: 15.53 ± 5.081 years and HC: 15.00 ± 2.89 years, p = 0.691) and gender (CD: 65% female, HC: 75% female, U= 180.00, *p* =.506).

### 4.2 Psychological distress

40% of participants with CD reported clinically elevated levels of depression and 60% reported clinically elevated anxiety.

95% of healthy controls scored within the non-clinical range for depression. 45% reported clinically elevated levels of anxiety. Mean, SD, and range of scores for participants with dystonia and HCs are displayed in Table 1.

**Table 1:** Mean (M), N, standard deviation (SD) and range of scores for depression/anxiety (HADS-A, HADS-D) for both groups.^1,2,3^

	Group	*N*	*M*	*SD*	Range	*Test Stat.*	*P*
Depression	CD	19	6.95	5.93	0–17		
	Control	20	3.80	2.44	0–9	*x^2^ = 7.557*	0.006*
Anxiety	CD	19	9.79	4.61	3–19		
	Control	20	7.85	3.41	3–14	*x^2^* = 2.092	0.148

* indicates statistically significant between-group differences.

The proportion of clinically significant depressive symptoms (HADS-D) was significantly higher for participants with CD than HCs (χ2 (1, N = 20) = 7.557, p =.006)). Despite higher mean symptoms of anxiety (HADS-A), the proportion of clinically significant anxiety symptoms (HADS-D) was not significantly different for participants with CD compared to healthy controls (χ2 (1, N = 20) = 2.092, p =.148)).

### 4.3 Social Cognition

**Fig. 1:**
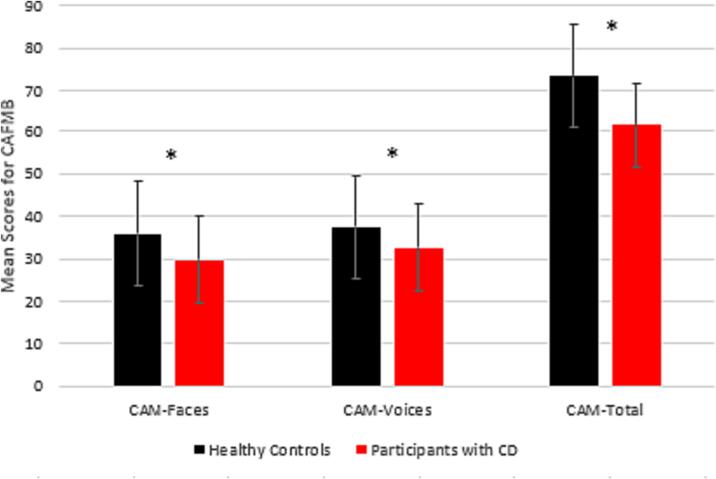
Mean Scores for CAMFB Subscales and Total Scores for both groups 1 * indicates statistically significant between-group differences. 2 All participants completed all tasks except two participants with CD who did not complete the ESCoT, and one who did not complete the HADS due to time constraints. 3 Error bars represent standard error of the represented means.

CAMFB: Participants with CD scored significantly lower than HCs on both subscales (CAM-Faces, CAM-Voices) and total CAMFB scores, indicating poorer recognition of emotional facial expressions and prosody by the participants with CD compared to HCs (Fig. 1).

ESCoT: Despite lower mean scores among the participants with CD, there was no significant difference between groups on cognitive theory of mind scores (Table 2). A MANOVA was conducted to explore between-groups differences on the remaining ESCoT subscales.

**Table 2:** Mean (M), N, standard deviation (SD) for subscales and total scores on SC measures for participant and control groups^1,2,3^.

	Group	*N*	*M*	*SD*	*Test Stat.*	*P*
CAM Faces*	CD	20	29.75	8.08		
	Control	20	35.95	6.83	*t*(38) = 2.621	0.013
CAM Voices*	CD	20	32.56	8.79		
	Control	20	37.45	5.69	*t*(38) = 2.093	0.043
CAMFB Total*	CD	20	61.60	16.88		
	Control	20	73.40	11.79	*t*(38) = 2.564	0.014
ESCoT: Cognitive ToM	CD	18	18.78	3.21		
	Control	20	19.95	1.76	*U* = 215	0.307
ESCoT: Affective ToM	CD	18	24.22	2.78		
	Control	20	24.50	2.98	*F*(1,36) = 0.088	0.769
ESCoT: Interpersonal understanding	CD	18	17.39	3.56		
	Control	20	16.70	2.94	*F*(1,36) = 0.428	0.517
ESCoT: Intrapersonal understanding	CD	18	22.78	3.52		
	Control	20	24.40	3.62	*F*(1,36) = 1.951	0.171
ESCoT: Total Score	CD	18	83.06	7.53		
	Control	20	84.90	7.59	*t*(36) = 0.750	0.458

* indicates statistically significant between-group differences.

**Fig. 2:**
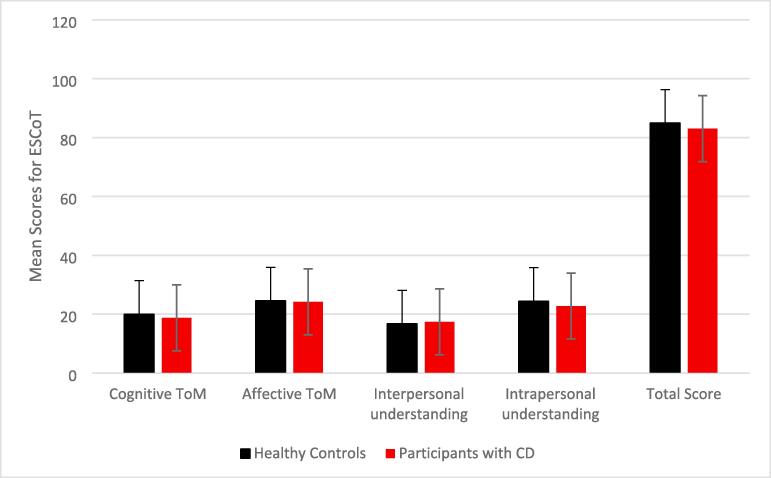
Mean Scores for ESCoT Subscales and Total Scores Across Groups. 1 * indicates statistically significant between-group differences. 2 All participants completed all tasks except two participants with CD who did not complete the ESCoT, and one who did not complete the HADS due to time constraints. 3 Error bars represent standard error of the represented means.

Using Pillai’s Trace, groups did not differ significantly on the overall combination of subscales (*F*(3,36) = 0.757, *p* =.526). Univariate analyses for each subscale also failed to reach statistical significance (Table 2). There was no significant difference between groups on total ESCoT scores (Fig. 2). Mean scores and SD for both groups on all measures of SC are displayed in Table 2.

## 5 Correlations

### 5.1 Social Cognitive, clinical and demographic measures

For the entire participant group, there was a significant strong positive correlation between anxiety (HADS-A) and depression (HADS-D) scores (*r* = 0.521, *p* = (0 0 1), indicating higher depressive scores were associated with higher anxiety scores, and vice versa. A significant moderate negative correlation between depression symptoms and CAM-Faces was observed (*r* = -0.231*, p* = 0.046), indicating higher symptoms of depression were associated with poorer understanding of emotional facial expressions.

There was a significant, moderate, positive correlation between years of education and recognition of emotional facial expressions (CAM-Faces) (*r* = 0.439, *p* =.005), prosody (CAM-Voices) (*r* = 0.489, *p* =.002) and affective theory of mind (ESCoT Affective ToM) (r = 0.329, *p* =.047), indicating an association between more years of education and greater ability to recognise emotions. There was also a significant, moderate negative correlation between age and scores on cognitive theory of mind (ESCoT cognitve ToM) (*r* = -0.416, *p* =.009), and CAM-Voices (*r* = -0.357, *p* =.024), indicating older age was associated with poorer performance on these subscales.

For all participants, there was a significant moderate correlation between cognitive theory of mind abilities (ESCoT cognitive ToM) and recognition of emotional facial expression (CAM-Faces) (*r =*.465*p =*.003)*,* and recognition of emotional prosody (CAM-voices) (*r =* 0.447*, p =*.005)*,* indicating that greater cognitive ToM abilities were associated with better recognition of complex emotions from visual and audio stimuli.

There was a significant positive correlation between scores of affective ToM (ESCoT) and recognition of emotional facial expressions (CAM-Faces) (*r =* 0.355, *p* =.029) and recognition of emotional prosody (CAM-Voices) (*r* = 0.364, *p* =.024) for all participants, indicating that better ability to infer the emotions of facial expressions and prosody was associated with greater ability to understand emotional states when observing a social interaction.

## 6 Discussion

Individuals with CD performed significantly worse than age- and sex-matched controls on scores of complex emotion recognition, as measured by the CAMFB [22]. Participants with CD did not differ significantly from controls in scores of any ESCoT subscale or total scores. The proportion of clinically significant depressive symptoms was significantly higher in participants with CD than controls. 40% of participants with CD reported clinically significant depression symptoms and 60% reported clinically significant symptoms of anxiety. Poorer understanding of emotional facial expressions was also associated with higher levels of depression in the CD group.

### 6.1 Our Findings in the context of previous research

Findings extends upon reports of basic emotion recognitions deficits for individuals with CD from visual and auditory stimuli [6], [7] and indicates this may extend to more complex emotional and mental states, using a measure novel to the CD literature. One study [5] indicated that most participants with CD performed adequately on an Affect Naming Task, based upon normative data. However, matched control group-based data has higher sensitivity compared to published normative data in detecting mild cognitive impairments [26]. The lack of significant between-group differences on inter- and intrapersonal understanding of social norms corresponds with previous research [5].

Our study contrasts with previous reports of poorer performance on cognitive ToM and affective ToM tasks for individuals with CD, compared to controls [13], [14]. This may be owing to cognitive variation across cohorts or the heterogenous array of study methods and assessment measures utilised across CD literature. Better cognitive ToM abilities have been associated with better scores on executive function tasks [5]. Mixed results regarding executive function in CD have been reported [2], [6]. Further, the assessment measure used, the ESCoT [20], has not been applied or validated for use in CD populations specifically, or may lack power to detect statistically significant differences owing to the sample size.

There is little consistency across the CD literature regarding ToM measures utilised, which makes direct comparison across studies challenging. Previous studies [5], [9], [13] have reported individuals experience difficulties in cognitive ToM, measured using the FP Test, a false belief reasoning task, and the Advanced Task. Conversely, participants with CD did not exhibit poorer cognitive ToM, in comparison to controls [6] or normative data [7] measured using the RMET. However, the RMET has been criticized as a measure of ToM [27]. Individuals with alexithymia and autism spectrum disorder (ASD), a cohort with well-established ToM deficits, score similarly on the RMET; however, participants with ASD score much lower on an alternate measure of ToM [27]. Many ToM tasks utilized in SC research are unsuitable for cohorts with co-morbid cognitive deficits due to information processing and comprehension demands [20]. This indicates a need for the development of appropriate and sensitive assessment socio-cognitive assessment measures, specific to adult-onset isolated focal dystonia (AOIFD). Notably, the efficacy of the CAMFB [22] in identifying significant differences between HCs and a small cohort of participants with CD may suggest its suitability for application in AOIFD and other neurological conditions.

Consistent with previous reports of psychological distress in CD cohorts [4] the proportion of clinically significant depressive symptoms was significantly higher for participants with CD than controls.

### 6.2 Theoretical applications

Observed findings support CD’s conceptualization as a network disorder manifesting in a syndrome with motor and non-motor symptomology. Poorer complex emotion recognition by the participants with CD compared to HCs support proposed collicular-pulvinar-amygdala pathway dysfunction in AOIFD [28].

### 6.3 Clinical applications

Poorer performance on socio-cognitive measures has significant implications for the assessment, treatment of, and research regarding CD. The association between observed poorer socio-cognitive performance in recognizing emotions from facial expressions and depressive symptoms indicates potential mood implications of socio-cognitive dysfunction. Including SC tasks to routine neuropsychological assessment may aid identification of subtle socio-cognitive difficulties. This may facilitate understanding of behavioral changes for friends and family of individuals with socio-cognitive difficulties and ease strain on important social relationships..

The observed group differences on depressive symptoms, association between socio-cognitive difficulties and depressive symptoms, and notable prevalence of psychological distress warrants monitoring for such symptoms in clinical practice. Psychological distress is a main predictor of health-related quality of life in CD [4]. Individuals with a diagnosis of AOIFD are twice as likely to be diagnosed with a depressive disorder than those without, and there are observed associations between depression and anxiety and suicide attempts/ deaths in AOIFD cohorts [29]. Greater levels of depressive symptoms have been associated with decreased empathy in patients in CD [5] and thus may additionally contribute to socio-cognitive difficulties.

### 6.4 Limitations & future research

A strength of this study is the application of novel, ecologically valid measures of SC to a CD cohort [21]. This present study is not without limitations. The sample of individuals with CD is small; CD is a rare and likely underdiagnosed condition which makes recruitment of large samples challenging. Participant recruitment took place during COVID-19. Patients reported less willingness to participate in hospital-based research at this time; which may have impacted recruitment for this study [29]. Thus, results should be considered with this in mind. Larger, multi-centre studies are warranted to further explore socio-cognitive dysfunction in CD.

Further, whilst participants with obvious cognitive deficits were excluded, the study did not control for co-morbid cognitive impairment amongst the CD cohort, or consider presence of tremor, which is associated with more severe cognitive ToM deficits [13]. Thus, further research is warranted to clarify whether SC difficulties are independent of broader cognitive deficits in CD, and to establish and validate more suitable assessment measures for CD populations.

## 7 Conclusion

Individuals with CD performed significantly worse than controls on a measure of complex emotion recognition. The proportion of clinically significant depressive symptoms was significantly higher for participants with CD than healthy controls. Given the potential quality of life impact of socio-cognitive dysfunction [15] and psychological distress [4] practitioners should routinely monitor psychological distress, socio-cognitive function and quality of life when working with individuals with CD.

## Declaration of Competing Interest

The authors declare that they have no known competing financial interests or personal relationships that could have appeared to influence the work reported in this paper.
